# Supplementary material for: Large-scale genomic analyses reveal insights into pleiotropy across circulatory system diseases and nervous system disorders
Source: Nat Commun. 2022 Jun 14;13:3428. doi: 10.1038/s41467-022-30678-w (PMC9198016; doi:10.1038/s41467-022-30678-w)
Supplement: Supplementary file 1 — Supplementary Information [file 41467_2022_30678_MOESM1_ESM.docx]

Supplementary Information for

Large-scale genomic analyses reveal insights into pleiotropy across circulatory system diseases and nervous system disorders

Xinyuan Zhang, Anastasia M. Lucas, Yogasudha Veturi, Theodore G. Drivas, William P. Bone,Anurag Verma, Wendy K. Chung, David Crosslin, Joshua C. Denny, Scott Hebbring, Gail P. Jarvik, Iftikhar Kullo, Eric B. Larson, Laura J. Rasmussen-Torvik, Daniel J. Schaid, Jordan W. Smoller, Ian B. Stanaway, Wei-Qi Wei, Chunhua Weng, Marylyn D. Ritchie

Correspondence to: [marylyn@pennmedicine.upenn.edu](mailto:marylyn@pennmedicine.upenn.edu%20)

**This PDF file includes:**

Supplementary Note

Supplementary Figures 1 to 6

**Other Supplementary Materials for this manuscript include the following:**

Supplementary Data 1 to 7

Supplementary Note

*Colocalization Analyses*

Colocalization analyses were performed on three of the five loci with evidence of pleiotropy– the other two loci (located near *HLA* region) were not analyzed due to complexity of the region. There is a possibility that there are separate signals driving the association in each pair of two traits, which might be due to LD and not pleiotropy. To address this, we performed colocalization analysis for the selected pairs of traits where we observed evidence of pleiotropy (one circulatory and one nervous system trait), aiming to determine if the same signal was driving both of the traits. We acknowledge that the posterior probabilities can be biased when we perform these analyses within the same cohort (since there will be sample overlap). We provide our results in the supplementary information and give the reader a reference. Among the three loci we tested, we do observe strong evidence for statistical colocalization (PP4 / (PP4 + PP3) >= 0.8) for all three loci we tested (Supplementary Fig. 7). The software *coloc* was used to perform these analyses^1^.

*Follow-up evaluation of the impact of phenotypic relationships*

The observation of pleiotropy between one SNP and multiple phenotypes can be potentially found in traits with comorbidity, which refers to the co-occurrence of two traits. Even though it is challenging to completely separate comorbidity and pleiotropy since they are interrelated, it is important to understand the extent and impact of comorbidity. To address this, we evaluated the proportion of overlapping cases as well as conducted conditional analyses on associated phenotypes of discovered pleiotropy.

For a phenotype pair that either belongs to the circulatory system or nervous system disease category, the top proportion of overlapping cases (>20%) are seen in phenotype pairs such as Alzheimer’s disease and dementia, atherosclerotic heart disease and angina pectoris, and among several circulatory system diseases (details are shown in Fig. S6). For phenotype pairs that individually belong to circulatory system and nervous system disease categories, however, we observed that the mean of proportion of overlapping cases is 3.76% in eMERGE and 4.22% in UKBB.

We evaluated the impact of the significance on SNP-phenotype associations by comparing the p-values to the p-values from analysis after adjusting for another phenotype in conditional analyses. We examined all pairwise combinations of phenotype of our discovered pleiotropy. We found a minimal impact on significance of phenotype pairs that had one circulatory disease and one nervous system disorder across all tested phenotypic pairs. Taken together with proportion of cases overlapping, our results suggest that our discovered pleiotropy across these two broad disease categories is likely from pleiotropic SNP-phenotype associations rather than due to comorbidity (Supplementary Fig. 6).

*Characterization of pleiotropy in sex-stratified analyses*

Sex differences have been described in cardiovascular diseases^78^ and neurological diseases^79^. We studied sex-specific pleiotropy by applying sex-stratified analyses on 607 SNPs which were identified across methods and datasets (see Results). We found three genomic regions that demonstrated pleiotropy from female-only and male-only analyses (Supplementary Table 6). Their associations with disease categories are shown in Supplementary Table 7 and Supplementary Fig. 5.

The identified SNPs on chromosome 19 near *APOE* showed risk pleiotropic associations with atherosclerotic heart disease, Alzheimer’s disease, and dementia in female and male, which was also seen in the combined analyses. However, the significant pleiotropic associations with delirium were only observed in male-only analyses. In addition, there are three SNPs in this region that showed protective effect on old myocardial infarction, or supraventricular tachycardia in females.

We have demonstrated in the combined analyses that SNPs near *CDKN2B-AS1* have opposite direction of genetic effect on a wide range of circulatory system diseases and depression (see Results). In females, most of the SNPs are associated with cerebral aneurysm and depression. In males, the SNPs are associated with a wide range of circulatory system diseases and delirium (with opposite direction of genetic effect) or bipolar disorders (with same direction of genetic effect), in addition to depression.

The SNP identified on chromosome 6 near *NOTCH4*;*LOC101929163* showed pleiotropic associations with essential hypertension and multiple sclerosis, which is observed from female-only analyses. The SNPs identified by male-only analyses located near *HLA-DRB1*;*HLA-DQA1* region showed opposite directions of genetic effect on coronary atherosclerosis and cardiovascular diseases, and bipolar disorders and multiple sclerosis. The association with bipolar disorders was not observed from combined analysis. There are two SNPs in this region (near *HLA-DRB1*;*HLA-DQA1*) that have demonstrated the same direction of genetic effect on diastolic heart failure and nervous system disorders from male-only analyses.

References

1. Giambartolomei C, *et al*. Bayesian Test for Colocalisation between Pairs of Genetic Association Studies Using Summary Statistics. PLoS Genetics, **15**; 10(5):e1004383, (2014).

**Supplementary Figures 1-6**

Supplementary Fig. 1. Study Analysis Plan

Overview of the analysis plan.

A B

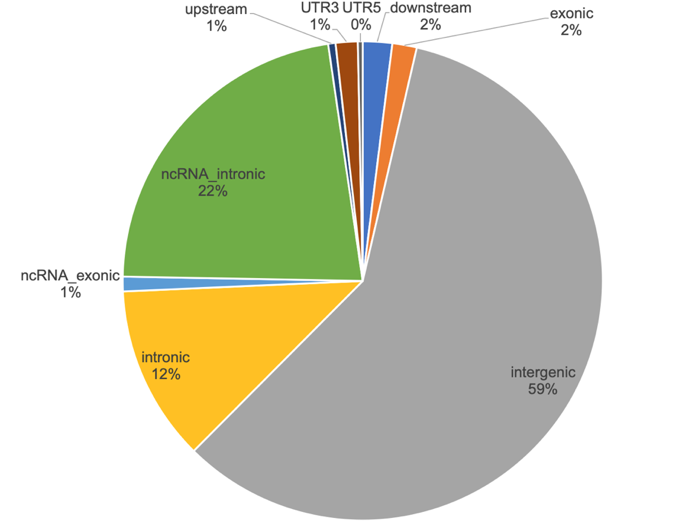


Supplementary Fig. 2. Functional annotations and overlap of SNPs identified by both PheWAS and MultiPhen

(A) Functional characteristics of 607 SNPs using ANNOVAR. (B) Comparison of the number of loci from applying PheWAS and MultiPhen on eMERGE and UKBB using Bonferroni significance threshold. The loci are counted when they suggest significant associations with at least one phenotype. For PheWAS, we included the loci when its minimum p-value among phenotypes passed the threshold. The Bonferroni significance are calculated as follows, eMERGE univariate: 0.05/7,642,122/147=4.45×10^-11^; UKBB univariate: 0.05/134,363/102=3.65×10^-9^; eMERGE multivariate: 0.05/7,642,122=6.54×10^-9^; UKBB multivariate: 0.05/134,363=3.72×10^-7^.

A B

****C D

Supplementary Fig. 3. Regional LD relationships around identified near pleiotropic loci.

(A) *CDKN2B-AS1* region on chromosome 9 from eMERGE. The phenotype in the top plot is coronary atherosclerosis of autologous vein bypass graft; and the bottom plot is major depressive affective disorder. (B) *HLA* region on chromosome 6 from eMERGE. The phenotype in the top plot is Parkinson’s disease; and the bottom plot is chronic pulmonary heart disease. (C) *HLA* region on chromosome 6 from eMERGE. The phenotype in the top plot is multiple scleorsis; and the bottom plot is pulmonary embolism and infarction. (D) *PRDM8* region on chromosome 4 from UKBB. The phenotype in the top plot is hypertension; and the bottom plot is severe depressive episode with psychotic symptoms.

A B


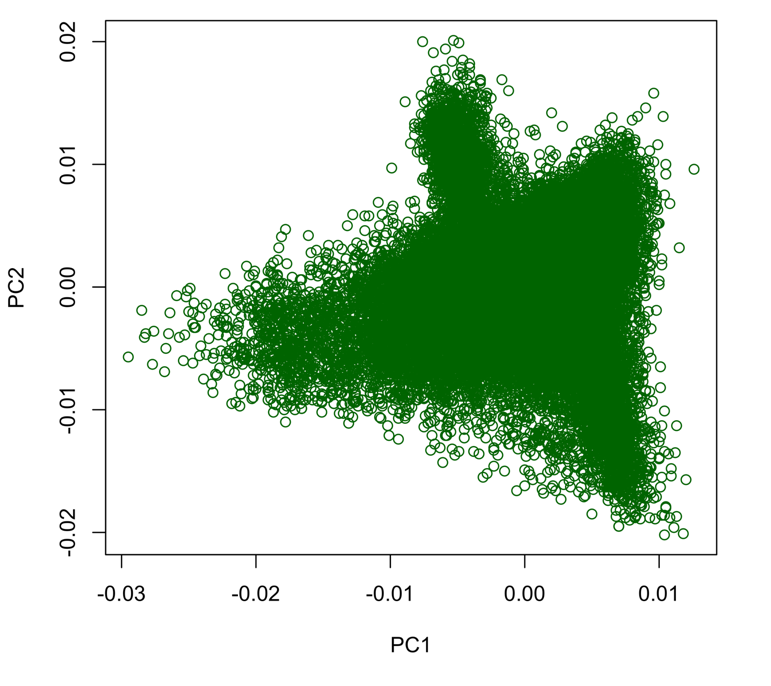

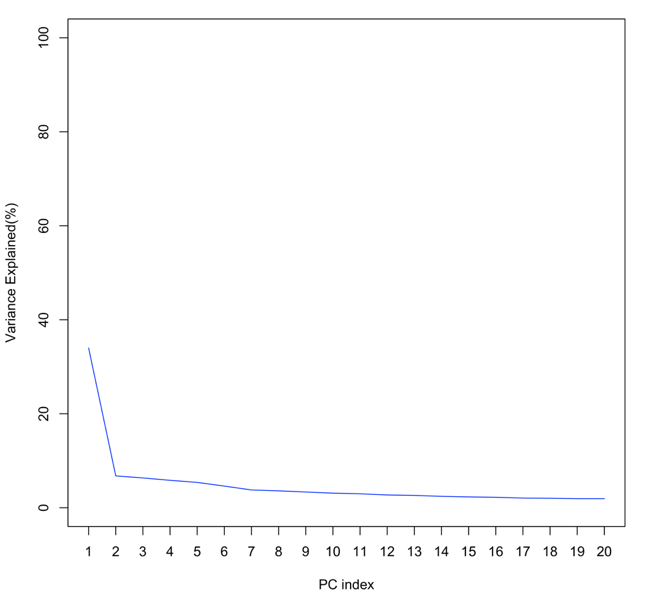


Supplementary Fig. 4. Principal components of ancestry in eMERGE

(A) PC1 versus PC2 on the European individuals included in this study from the eMERGE network. (B) Proportion of variance explained by the top 20 PCs in the eMERGE network.

 A B

Supplementary Fig. 5. Characterization of pleiotropy from sex-stratified analyses.

(A) Female-only analysis; (B) Male-only analysis. The results are obtained from sequential multivariate analyses from both eMERGE network and UK Biobank.


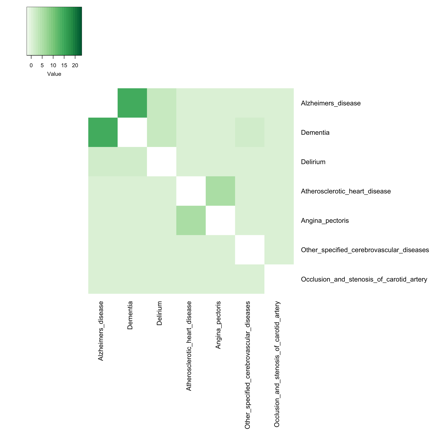

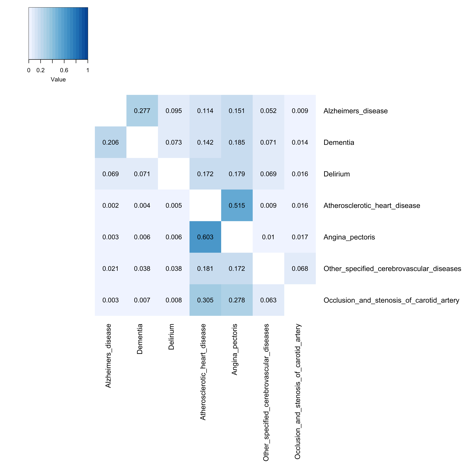
A


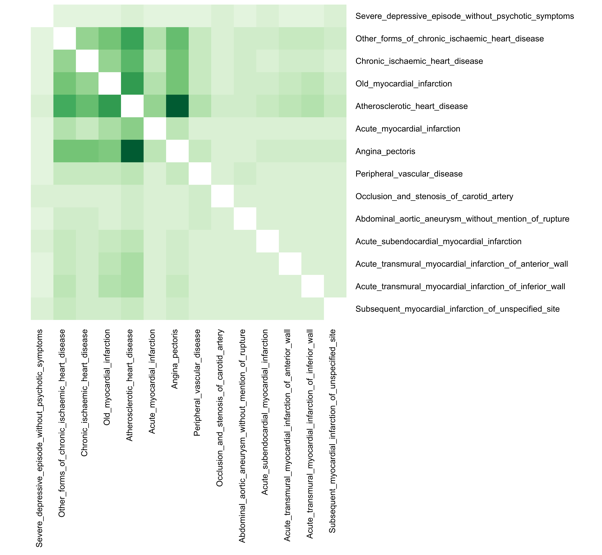

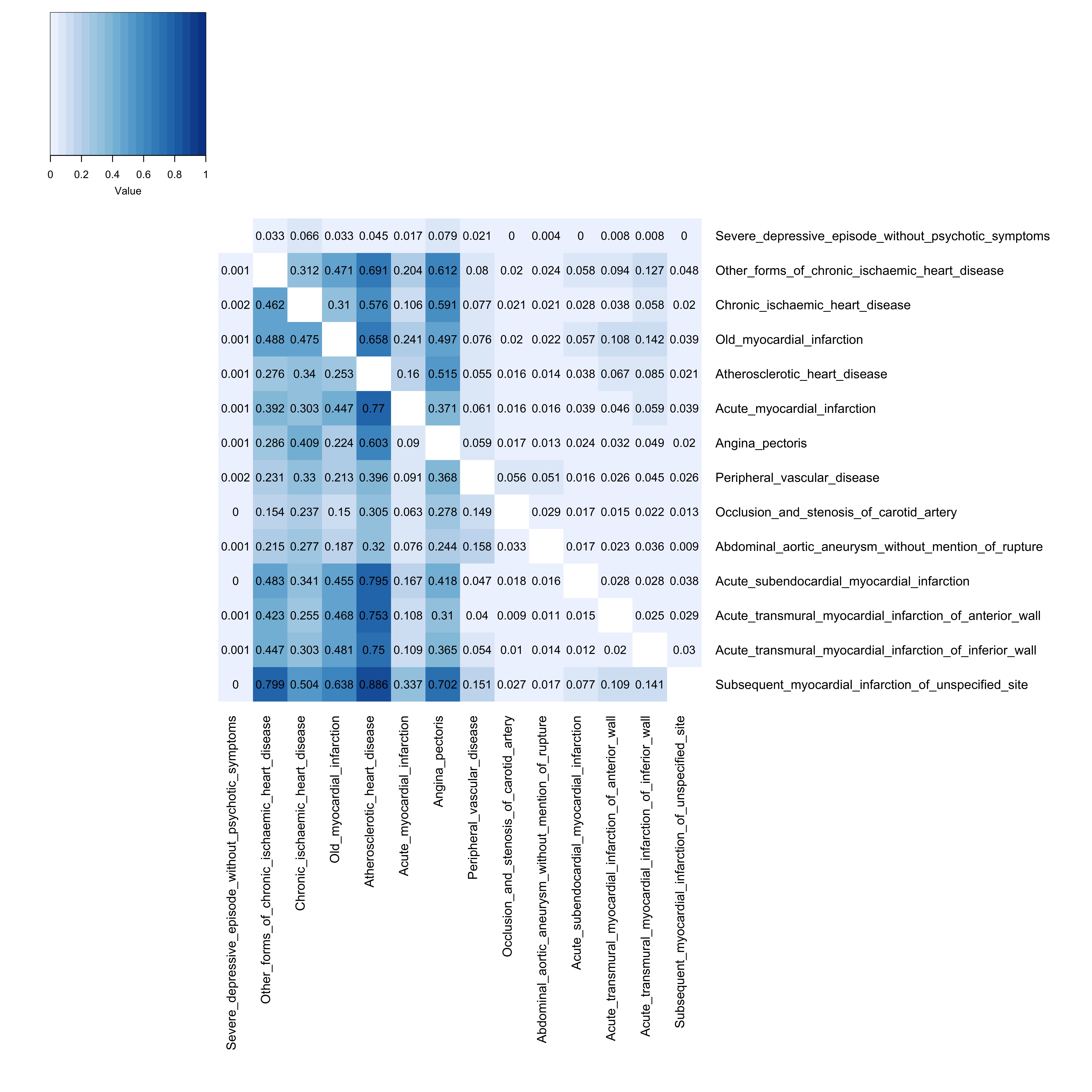
B


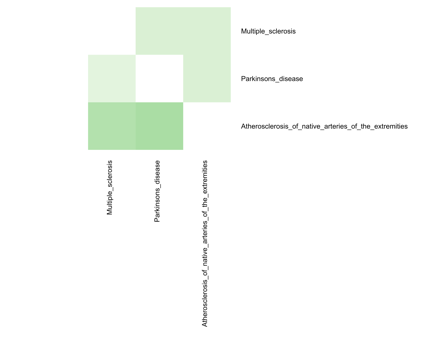

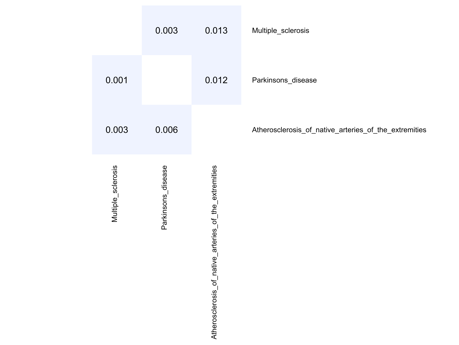


C


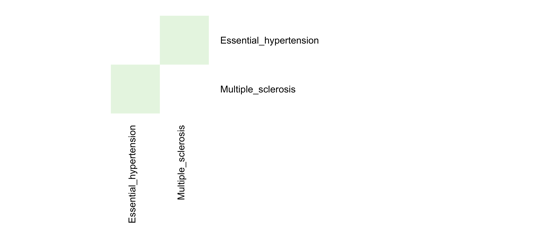


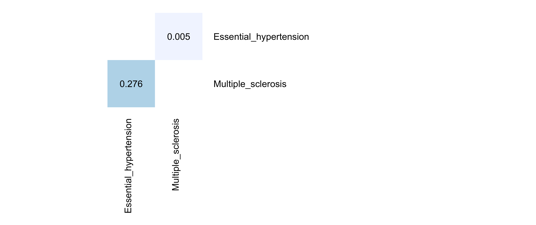


D


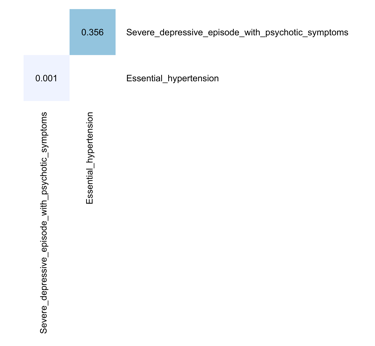


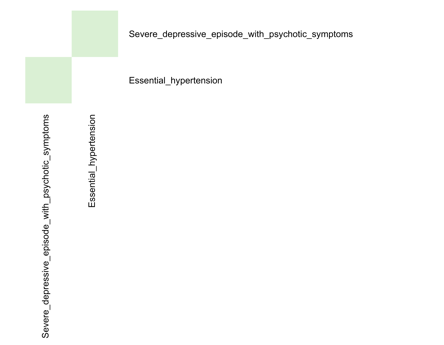
E

Supplementary Fig. 6. Evaluation of case overlap and effect sizes on each identified phenotype set with evidence for pleiotropy

The proportion of case overlap (the left plot) and the mean of log odds ratio between conditional analyses and independent analyses (the right plot). (A) *APOE* region on chromosome 19 from UKBB. (B) *CDKN2B* region on chromosome 9 from UKBB. (C) *HLA* region on chromosome 6 from eMERGE. (D) *NOTCH4* region on chromosome 6 from UKBB. (E) *PRDM8* region on chromosome 4 from UKBB.

SNP rs16998073:


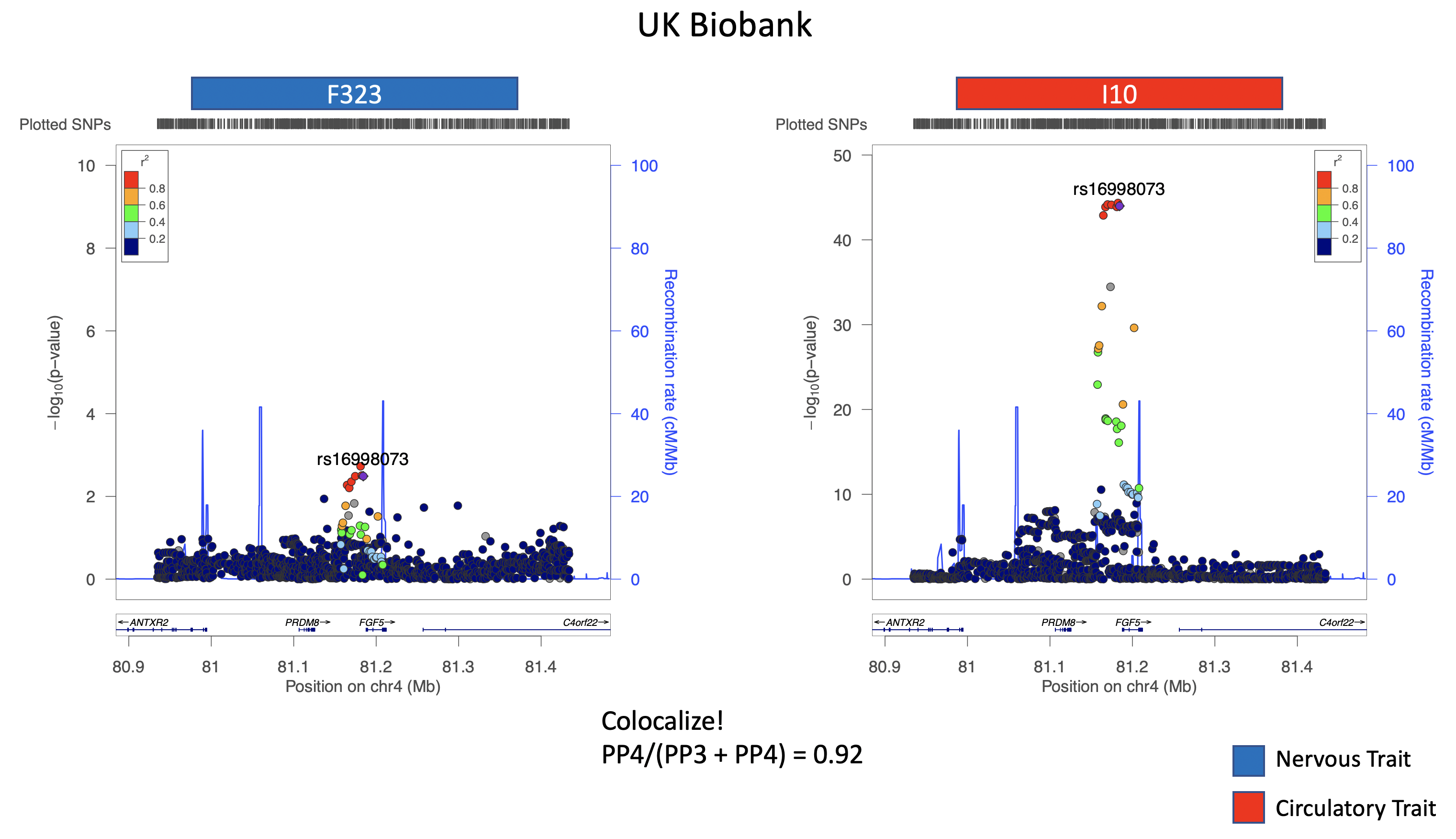


SNP rs157582:


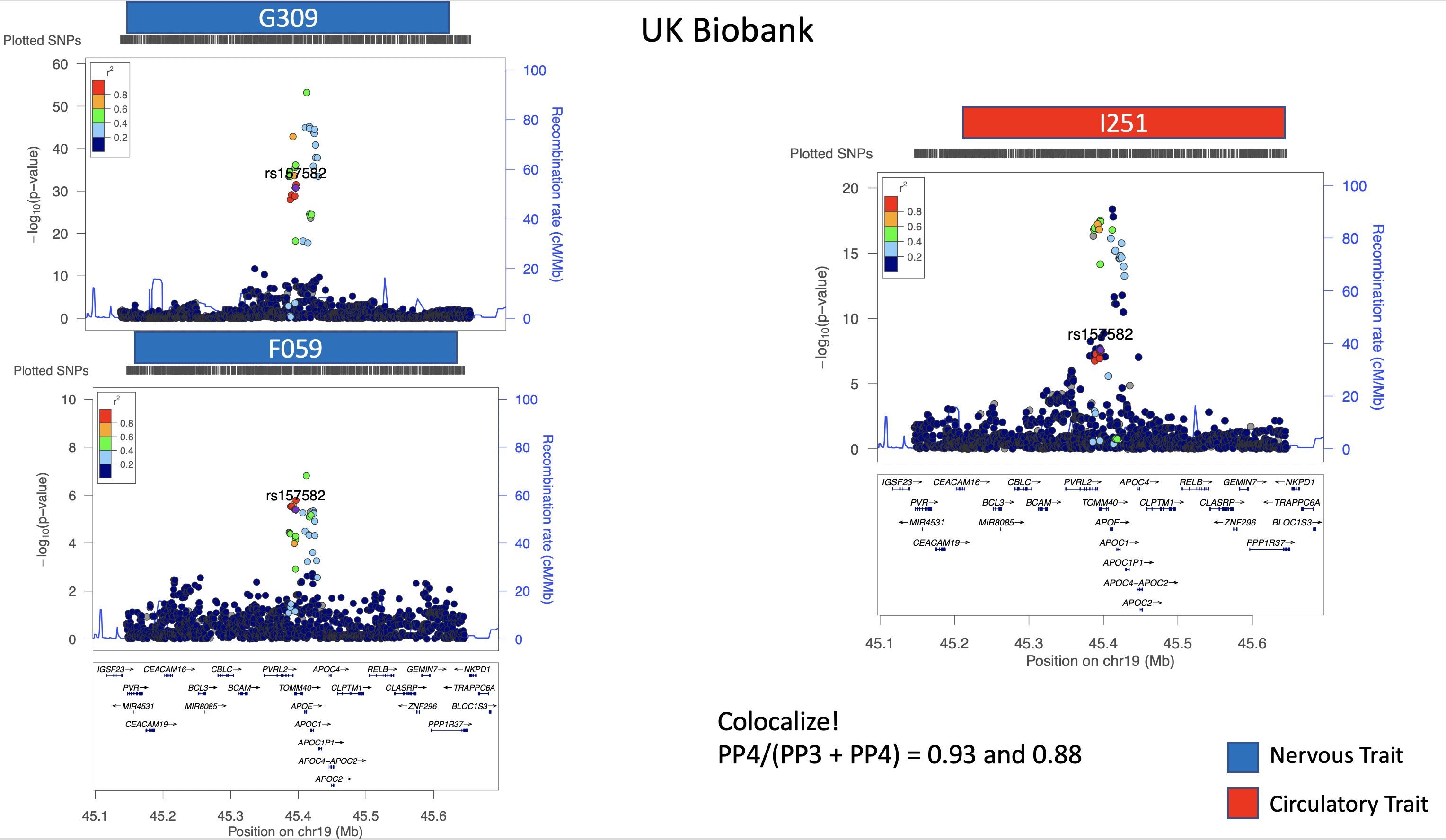


SNP rs10811656:


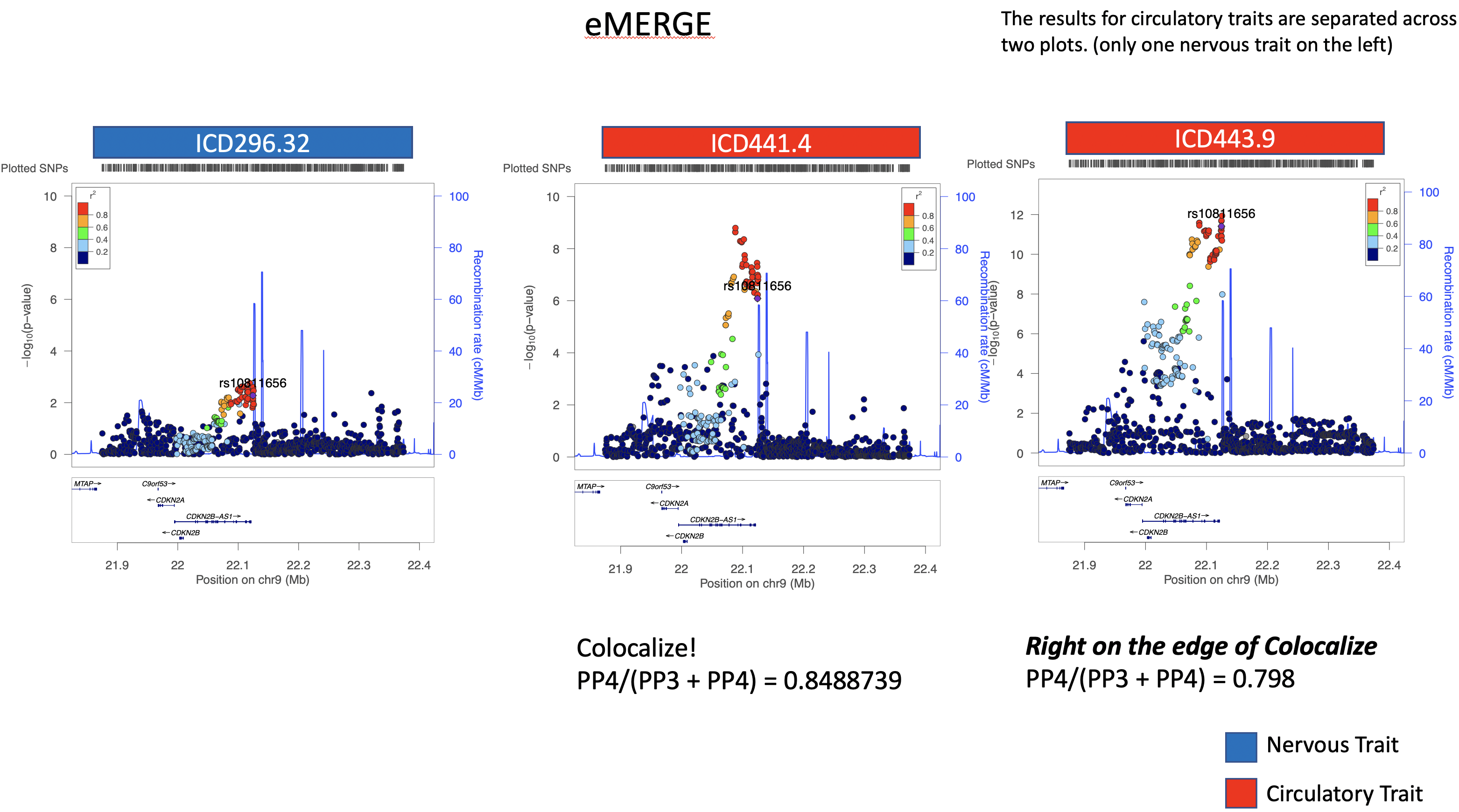


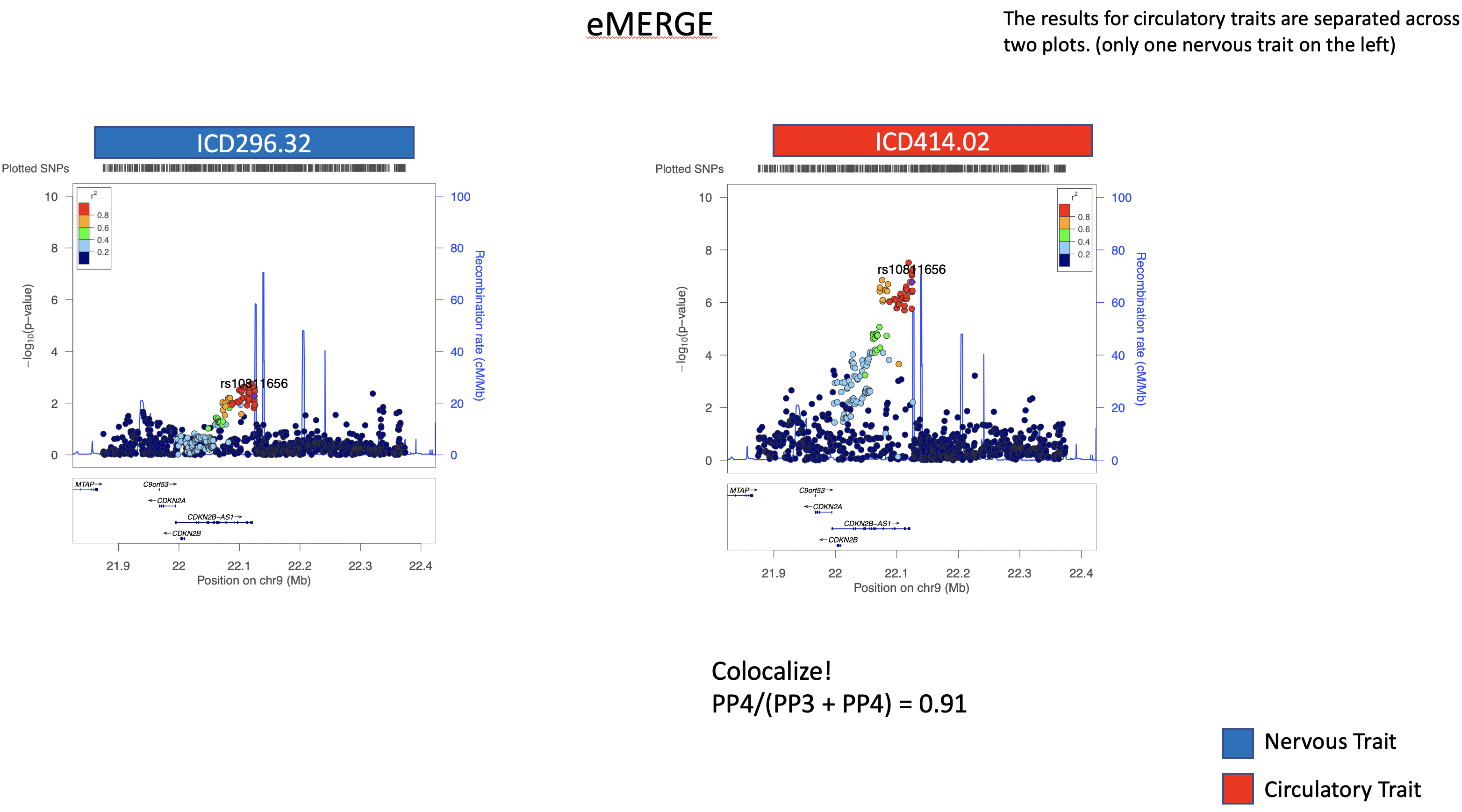


**Supplementary Fig. 7.** **Colocalization results on three identified loci.**

The comparison between the pair of traits is within the same dataset denoted in the title.
