# Supplementary material for: Large-scale genomic analyses reveal insights into pleiotropy across circulatory system diseases and nervous system disorders
Source: Nat Commun. 2022 Jun 14;13:3428. doi: 10.1038/s41467-022-30678-w (PMC9198016; doi:10.1038/s41467-022-30678-w)
Supplement: Supplementary file 3 — Description of Additional Supplementary Files [file 41467_2022_30678_MOESM3_ESM.pdf]

Description of Additional Supplementary Files

File Name: Supplementary Data 1
Description: Circulatory system diseases and nervous system disorders that were included in this study. This table provided ICD codes, case sample size and disease group in eMERGE and UKBB.

File Name: Supplementary Data 2
Description: Genetic variants that were identified by both PheWAS and MultiPhen from both eMERGE and UKBB. This table provided the rsID, genomic location on chromosome (GRCh37/hg19), functional annotation, mapped genes and LD pruned SNPs.

File Name: Supplementary Data 3
Description: Discovered pleiotropy and information from GWAS catalog. This table provided the pleiotropic SNPs, direction of genetic effect size and their associated phenotypes. It also provided the association of the SNPs with circulatory or nervous system related traits from GWAS catalog.

File Name: Supplementary Data 4
Description: Univariate summary statistics for discovered pleiotropic regions in the eMERGE and UKBB.

File Name: Supplementary Data 5
Description: Pleiotropic genes and their associated disease categories in eMERGE and UKBB.

File Name: Supplementary Data 6
Description: Discovered pleiotropy from sex-stratified analyses. This table provided the rsID, associated phenotypes and the direction of genetic effect size.

File Name: Supplementary Data 7
Description: Pleiotropic genes obtained from sex-stratified analyses and their associated disease categories in eMERGE and UKBB.
