# Supplementary material for: Large-scale genomic analyses reveal insights into pleiotropy across circulatory system diseases and nervous system disorders
Source: Nat Commun. 2022 Jun 14;13:3428. doi: 10.1038/s41467-022-30678-w (PMC9198016; doi:10.1038/s41467-022-30678-w)
Supplement: Supplementary file 11 — Reporting Summary [file 41467_2022_30678_MOESM11_ESM.pdf]

nature research

Corresponding author(s): Marylyn D. Ritchie

Last updated by author(s): February 14, 2022

Reporting Summary

Nature Research wishes to improve the reproducibility of the work that we publish. This form provides structure for consistency and transparency in reporting. For further information on Nature Research policies, see our Editorial Policies and the Editorial Policy Checklist.

Statistics

For all statistical analyses, confirm that the following items are present in the figure legend, table legend, main text, or Methods section.

n/a	Confirmed	
☐	☒	The exact sample size (*n*) for each experimental group/condition, given as a discrete number and unit of measurement
☐	☒	A statement on whether measurements were taken from distinct samples or whether the same sample was measured repeatedly
☐	☒	The statistical test(s) used AND whether they are one- or two-sided<br>*Only common tests should be described solely by name; describe more complex techniques in the Methods section.*
☐	☒	A description of all covariates tested
☐	☒	A description of any assumptions or corrections, such as tests of normality and adjustment for multiple comparisons
☐	☒	A full description of the statistical parameters including central tendency (e.g. means) or other basic estimates (e.g. regression coefficient) AND variation (e.g. standard deviation) or associated estimates of uncertainty (e.g. confidence intervals)
☐	☒	For null hypothesis testing, the test statistic (e.g. *F*, *t*, *r*) with confidence intervals, effect sizes, degrees of freedom and *P* value noted<br>*Give P values as exact values whenever suitable.*
☒	☐	For Bayesian analysis, information on the choice of priors and Markov chain Monte Carlo settings
☐	☒	For hierarchical and complex designs, identification of the appropriate level for tests and full reporting of outcomes
☒	☐	Estimates of effect sizes (e.g. Cohen's *d*, Pearson's *r*), indicating how they were calculated

Our web collection on statistics for biologists contains articles on many of the points above.

Software and code

Policy information about availability of computer code

Data collection	No software was used in this manuscript during data collection stage. We used PLINK 1.9 for quality control.
Data analysis	PLINK 1.9; PLATO 2.1.0; MultiPhen R package 2.0.2; Pleio R package 1.6; Hudson R package (only one version available); Coloc R package 3.2

For manuscripts utilizing custom algorithms or software that are central to the research but not yet described in published literature, software must be made available to editors and reviewers. We strongly encourage code deposition in a community repository (e.g. GitHub). See the Nature Research guidelines for submitting code & software for further information.

Data

Policy information about availability of data

All manuscripts must include a data availability statement. This statement should provide the following information, where applicable:

- Accession codes, unique identifiers, or web links for publicly available datasets
- A list of figures that have associated raw data
- A description of any restrictions on data availability

This project is under UK Biobank application ID 32133. The dbGaP study accession number for eMERGE Network Phase III is phs001584.v1.p1. Both eMERGE and UK Biobank data are controlled access data and would be available upon application to either the UK Biobank or dbGaP, respectively. The data are controlled access due to privacy laws.

Field-specific reporting

Please select the one below that is the best fit for your research. If you are not sure, read the appropriate sections before making your selection.

☒ Life sciences ☐ Behavioural & social sciences ☐ Ecological, evolutionary & environmental sciences

For a reference copy of the document with all sections, see nature.com/documents/nr-reporting-summary-flat.pdf

Life sciences study design

All studies must disclose on these points even when the disclosure is negative.

Sample size	The total sample size of each biobank was predetermined by the eMERGE network and the UK Biobank. We applied a case number threshold of 200 for each phenotype to select the phenotypes to include in our study, according to a previously published simulation study. See details about sample size in manuscript and supplementary table 1.
Data exclusions	We excluded samples that failed quality control procedure (See details in Genotype Quality Control section in 'Methods').
Replication	We performed our analysis pipeline in two independent cohorts, including the application of independent statistical methods.
Randomization	This is not relevant to our study since it is not a randomized controlled trail.
Blinding	This is not relevant to our study since it is not a randomized controlled trail.

Reporting for specific materials, systems and methods

We require information from authors about some types of materials, experimental systems and methods used in many studies. Here, indicate whether each material, system or method listed is relevant to your study. If you are not sure if a list item applies to your research, read the appropriate section before selecting a response.

Materials & experimental systems

n/a	Involved in the study	
☒	☐	Antibodies
☒	☐	Eukaryotic cell lines
☒	☐	Palaeontology and archaeology
☒	☐	Animals and other organisms
☐	☒	Human research participants
☒	☐	Clinical data
☒	☐	Dual use research of concern

Methods

n/a	Involved in the study	
☒	☐	ChIP-seq
☒	☐	Flow cytometry
☒	☐	MRI-based neuroimaging

Human research participants

Policy information about studies involving human research participants

Population characteristics	We studied circulatory system diseases and neurological disorders in European population from the eMERGE network and the UK Biobank. We studied common variants from the imputed genotype data from both datasets. For eMERGE, we studied individuals with age larger or equal to 25, and about 50% female and 50% male. The age of the samples in the UK Biobank is larger than 40 and also have about 50% female and 50% male.
Recruitment	The participants were recruited by eMERGE consortium and the UK Biobank.
Ethics oversight	Research conducted in this study complies with all ethical regulations laid out in the Declaration of Helsinki. This study was performed in the eMERGE Network, which is a funded consortium sponsored by the National Human Genome Research Institute (NHGRI) that combined biorepositories with EHR data across leading medical institutions. All studies were approved by the institutional review boards of each respective institution. Each participant gave consent for being part of the DNA biobanks. Data from the UK Biobank for this project pertained to application no. 32133.

Note that full information on the approval of the study protocol must also be provided in the manuscript.
